# Nurses’ worry or concern and early recognition of deteriorating patients on general wards in acute care hospitals: a systematic review

**DOI:** 10.1186/s13054-015-0950-5

**Published:** 2015-05-20

**Authors:** Gooske Douw, Lisette Schoonhoven, Tineke Holwerda, Getty Huisman-de Waal, Arthur R H van Zanten, Theo van Achterberg, Johannes G van der Hoeven

**Affiliations:** Gelderse Vallei Hospital, Willy Brandtlaan 10, Ede, 6716, RP The Netherlands; University of Southampton, Level A, (MP11) South Academic Block, General Hospital, Tremona Road, Southampton, SO16 6YD UK; Radboud University Medical Centre, Radboud Institute for Health Sciences, IQ Healthcare, Postbus 9101, Nijmegen, 6500, HB The Netherlands; Centre for Health Services and Nursing Research, KU Leuven, Kapucijnenvoer 35 blok d - box 7001, 3000 Leuven, Belgium; Department of Intensive Care, Radboud university medical centre, Postbus 9101, Nijmegen, 6500 HB The Netherlands

## Abstract

**Introduction:**

Nurses often recognize deterioration in patients through intuition rather than through routine measurement of vital signs. Adding the ‘worry or concern’ sign to the Rapid Response System provides opportunities for nurses to act upon their intuitive feelings. Identifying what triggers nurses to be worried or concerned might help to put intuition into words, and potentially empower nurses to act upon their intuitive feelings and obtain medical assistance in an early stage of deterioration. The aim of this systematic review is to identify the signs and symptoms that trigger nurses’ worry or concern about a patient’s condition.

**Methods:**

We searched the databases PubMed, CINAHL, Psychinfo and Cochrane Library (Clinical Trials) using synonyms related to the three concepts: ‘nurses’, ‘worry/concern’ and ‘deterioration’. We included studies concerning adult patients on general wards in acute care hospitals. The search was performed from the start of the databases until 14 February 2014.

**Results:**

The search resulted in 4,006 records, and 18 studies (five quantitative, nine qualitative and four mixed-methods designs) were included in the review. A total of 37 signs and symptoms reflecting the nature of the criterion worry or concern emerged from the data and were summarized in 10 general indicators. The results showed that worry or concern can be present with or without change in vital signs.

**Conclusions:**

The signs and symptoms we found in the literature reflect the nature of nurses’ worry or concern, and nurses may incorporate these signs in their assessment of the patient and their decision to call for assistance. The fact that it is present before changes in vital signs suggests potential for improving care in an early stage of deterioration.

**Electronic supplementary material:**

The online version of this article (doi:10.1186/s13054-015-0950-5) contains supplementary material, which is available to authorized users.

## Introduction

Early recognition and treatment of critically ill patients in general wards is a key aspect of Rapid Response Systems (RRSs). The aim of RRSs is to reduce intensive care unit (ICU) admissions, length of ICU stay, hospital length of stay and mortality [1].

Nurses often recognize patients in the ward who are deteriorating through intuition rather than through routine measurement of vital signs [2]. Intuition is an ability to understand or know something immediately based on feelings rather than facts [3]. In nursing research, Benner *et al.* [4] define intuition as ‘a judgment without a rationale, a direct apprehension and response without recourse to calculative rationality’. Nurses develop this skill over time, and often anticipate a patient’s decline before any objective evidence of deterioration is present [4].

The activation of an RRS is usually based on the recording of vital signs that deviate from predetermined values [5, 6]. Respiratory rate, oxygen saturation, heart rate, blood pressure, temperature and consciousness are often included, but in addition to these objective criteria, the subjective criterion ‘nurses’ worry or concern’ may be important [7, 8]. It provides an opportunity for nurses to call assistance when they intuitively feel that something is wrong with a patient, even when vital signs do not (yet) meet RRS calling criteria. However, RRSs value this criterion differently. Worry or concern can be a single calling criterion, in which case the team can be activated based solely on worry or concern [9]. This provides optimal opportunities for nurses to act upon their intuitive feelings and get assistance in an early stage of deterioration. In the combined approach, subjective criteria like worry or concern are added to objective criteria in an aggregated system [10]. This reduces possibilities for nurses to activate an RRS in an early stage, since vital signs must also be deteriorating. In RRSs that do not include the worry or concern criterion, it can be harder for nurses to get assistance when objective evidence is lacking [11, 12].

So far it is unclear whether including worry or concern as a calling criterion results in better patient outcomes. We need a better understanding of its essence. Identifying what triggers nurses’ worry or concern might help nurses to put intuition into words, and potentially empower them to act upon their intuitive feelings and obtain medical assistance for the patient in an early stage of deterioration. The aim of this systematic review is to identify the signs and symptoms that trigger nurses’ worry or concern about a patient’s condition.

## Methods

A systematic review of quantitative and qualitative studies was performed using the systematic review guidelines from the ‘Centre for Reviews and Dissemination’ [13] as guidance to structure the review process.

### Selection criteria

We included full-text original studies (all designs and languages), performed on general wards (adult patients, aged 18 years and older) in acute care hospitals, addressing the worry or concern of nurses in the process of recognition of deterioration in patients, or preceding calling for assistance and/or activation of the Rapid Response Team (RRT). We excluded studies that focused solely on specialized wards, such as emergency departments, ICUs, medium care units, obstetrics wards, operating rooms, pediatric wards and psychiatry wards, or studies concerning homecare. We also excluded studies of low methodological quality (see Quality appraisal). A table with the selection criteria is presented in Additional file 1.

### Search strategy

**Fig. 1:**
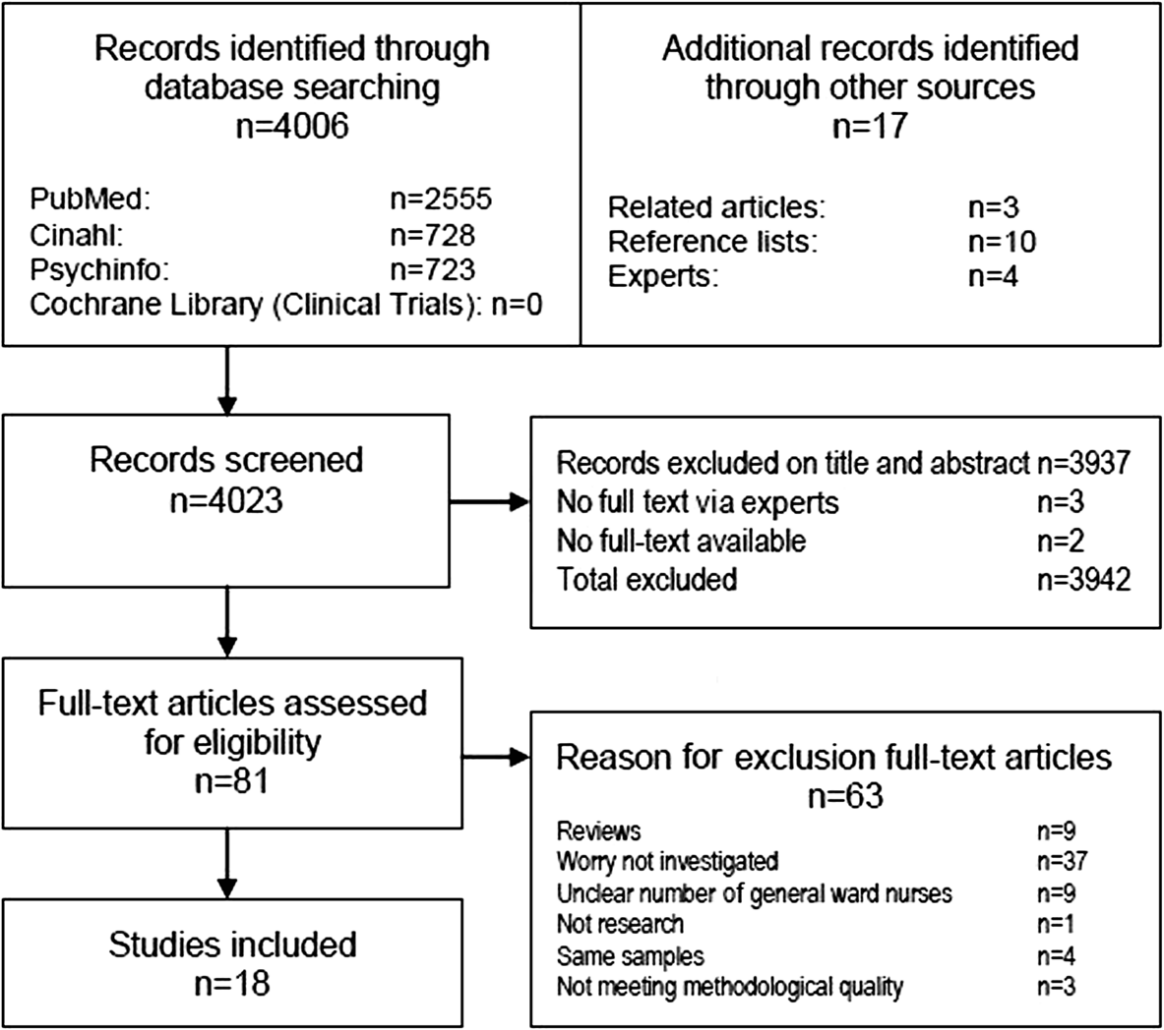
Flow diagram of the selection procedure

First, we searched the databases PubMed, CINAHL, Psychinfo and Cochrane Library (Clinical Trials) for original studies. We combined three major search terms: ‘nurses’, ‘worry/concern’ and ‘deterioration’. Synonyms for these search terms were also used, which can be found in the complete PubMed search presented in Additional file 2. We used a two-stage study selection for the database search: an initial screening of titles and abstracts against inclusion criteria and assessment of the full-text articles of potentially eligible studies. The search was performed from the start of the databases until 14 February 2014. Second, experts on the subject were asked for unpublished studies. Third, studies included for full-text reading were used to locate related articles using the ‘related citations’ link of the databases. Finally, references of included articles were examined for additional studies. Figure 1 gives a complete overview of the search strategy.

### Quality appraisal

We used the Strengthening the Reporting of Observational Studies in Epidemiology (STROBE) instrument [14] to assess quantitative study quality. Included items were: design, eligibility criteria, selection procedure, outcomes, risk of bias, study size, number and characteristics of participants, statistical methods, relevant subgroups and results. We valued items as positive, negative or unclear. Studies with between nine and 11 positive scores were considered to be of high methodological quality, those with between five and eight positive scores to be of moderate quality and those with less than five positive scores to be of low methodological quality.

Qualitative and mixed-methods studies were assessed using the National Institute for Health and Clinical Excellence Methodology checklist: qualitative studies [15]. This tool has six sections: theoretical approach, study design, data collection, validity, analysis and ethics. An overall score of quality is not included as not all measurement domains are considered equally important [16]. The assessment was used to gain understanding of relative strengths and weaknesses of eligible studies.

### Data extraction

We extracted the following data: design, aim, data collection, sample, setting, RRS (calling) system and outcomes. Outcomes extracted were the signs and symptoms underlying worry or concern of nurses.

### Review process

The database search (GD and LS), data selection (GD and LS), methodological quality assessment (TvA, GD and TH) and data extraction (GD and LS) were independently performed by two researchers. Disagreement was solved through discussion, and a third researcher (TvA or AvZ) was available in case of doubt.

### Synthesis

We included heterogeneous studies and as such, a meta-analysis could not be performed. Since our aim is strictly explorative, analysis of the data from both quantitative and qualitative studies was undertaken. Two researchers (GD and TH) independently analyzed all signs and symptoms that were extracted from the literature and separately suggested the themes that emerged from the data. The indicators were determined (GD and TH) through discussion and presented to three researchers (LS, TvA and AvZ) for agreement. Disagreement was solved through discussion until consensus was reached.

## Results

### Search outcome

The database search provided 4,006 records. One additional article and three abstracts of congress (poster) presentations were retrieved via experts. Additionally, three articles were retrieved via ‘related articles’ in the databases, and 10 articles via reference lists of the included studies. In total, 3,937 articles of the database search did not meet the inclusion criteria. Two articles from the reference lists were not available, and there were no articles on the three congress abstracts. The full-text of 81 publications was examined; 56 were excluded as they did not meet the selection criteria and three studies were excluded for low methodological quality [17–19]. Of the remaining 22 publications [11, 20–40], four additional studies were removed because of overlapping results in the same patient samples [25, 28, 29, 32]. This resulted in 18 studies included in the review (Fig. 1).

### Quality assessment

Quality assessment of the quantitative studies resulted in one high [24], four moderate [20–23] and three low quality studies [17–19]. The low quality studies were excluded. The qualitative studies [11, 26–40] had several limitations. However, as described in the methods section, they were all included. Detailed information of the quality assessment is presented in Tables 1 and 2.

**Table 1:** Quality assessment of quantitative studies

First author	Year	Reference	Are objectives clearly stated?	Is the design appropriate?	Are eligibility criteria, sources and methods of selection of participants described?	Are outcomes described?	Sources of data and details about methods of measurements described appropriate?	Risk of bias taken into account?	Is the study size adequate?	Are characteristics, numbers of participants and reasons for non-participation described?	Is the statistical method adequate?	Are relevant subgroups described in results?	Are results properly described?	Overall study quality
Bertaut	2008	[17]	-	-	-	-	-	-	?	-	-	?	+	L
Boniatti	2010	[20]	+	±	+	+	-	±	+	+	+	?	+	M
Hourihan	1995	[21]	+	+	+	+	+	+	±	+	+	±	+	M
Laurens	2011	[22]	±	±	±	±	±	+	±	+	+	+	+	M
Offner	2007	[18]	+	±	±	+	-	-	?	±	+	?	+	L
Parr	2001	[23]	+	+	+	+	±	?	+	+	+	?	+	M
Santiano	2009	[24]	+	+	+	+	±	+	+	+	+	+	+	H
Thomas	2007	[19]	-	?	-	?	-	?	?	+	+	+	?	L

+ = yes; ± = partly; − = no; ? = not assessable; H = high; L = low; M = moderate

**Table 2:** Quality assessment of qualitative studies

First author, year	Reference	Qualitative approach appropriate?	Aim/literature/theory	Study design	Data collection methods	Validity: Role researcher	Validity: Description context	Validity: Reliability methods	Validity: Rigorous+ /not rigorous - /not sure or not reported ±	Analysis: Rich+ /poor - /not sure or not reported ±	Analysis: Reliable+ /unreliable - / not sure or not reported ±	Analysis: Findings convincing	Analysis: Findings relevant to aim of study	Analysis: Conclusion adequate	Ethical considerations
		Appropriate +/inappropriate - /not sure ±	Clear +/unclear- /mixed ±	Defensible +/not defensible - /not sure ±	Appropriate+ /inappropriate - /not sure ±	Clear+ /unclear - /not described ±	Clear+ /unclear- /not sure ±	Reliable/unreliable - /not sure ±				Convincing+ /not convincing - /not sure ±	Relevant+ /irrelevant - /partially relevant ±	Adequate+ /inadequate - / not sure ±	Clear+ /unclear - /not sure or not reported ±
Andrews, 2005	[11]	+	+	±	-	±	±	±	+	+	±	±	+	+	+
Cioffi, 2009	[26]	±	+	±	+	±	+	±	±	±	±	+	+	+	+
Cioffi, 2000	[27]	+	+	+	+	±	+	±	±	+	+	+	+	±	±
Cox, 2006	[30]	+	±	±	±	+	±	-	+	+	±	+	+	+	+
Donaldson, 2009	[31]	+	+	+	±	±	±	+	+	-	+	±	+	±	-
Endacott, 2007	[33]	+	±	±	+	±	+	+	±	±	±	+	+	±	+
Gazarian, 2010	[34]	+	+	+	±	±	+	±	±	+	±	+	+	+	±
Leach, 2010	[35]	+	±	+	±	±	±	±	+	±	±	-	+	+	-
Massey, 2014	[36]	+	+	+	+	+	+	+	+	±	±	+	+	±	+
McDonnell, 2013	[37]	+	+	+	+	-	+	±	±	±	±	+	+	±	+
Minick, 2003	[38]	+	+	+	±	±	+	+	+	+	±	+	+	+	±
Pattison, 2011	[39]	+	+	+	+	±	±	±	+	+	±	+	+	+	+
Williams, 2011	[40]	+	+	+	+	+	+	±	+	+	±	+	+	+	+

### Characteristics of included studies

We found large heterogeneity in the studies, including in design. Studies were conducted in Australia (n = 8), the US (n = 5), the UK (n = 4) and Brazil (n = 1), with hospital settings varying from peripheral (non) teaching hospitals to university hospitals. Six studies included all wards, four included general wards and four studies were performed on medical wards. Four studies that analyzed RRS calls did not specify wards, but were included since the description in the articles suggest that general wards were involved. Studies comprised data on nurses (n = 13), of which five studies also included physicians and/or other healthcare workers. Worry or concern was the primary endpoint in five studies [26, 27, 33, 34, 39].

Five studies had quantitative designs: one quasi-experimental design [22] and four observational studies [20, 21, 23, 24]. Nine studies had qualitative designs: two grounded theory [11, 35], one phenomenology [38], one interpretative [36] and five descriptive studies [26, 27, 30, 34, 40]. We retrieved four mixed-methods studies, of which the qualitative part was relevant for the review [31, 33, 37, 39].

A total of 12 studies reported on RRSs: seven Medical Emergency Teams (all in Australia), with single-parameter calling systems, of which six included worry as a calling criterion and one study did not specify; three outreach teams (all in the UK) with aggregated calling systems without worry as calling criterion; and two RRTs (in the US) (one nurse-led) made no mention of the type of calling system. A summary of study characteristics is shown as Additional file 3.

### Signs and symptoms underlying worry or concern

A total of 170 signs and symptoms were extracted from the included articles that describe worry or concern (Table 3). For synonyms, one major term was chosen, reducing the 170 terms to 37 different signs and symptoms. These 37 signs and symptoms were categorized into 10 general indicators: change in respiration, change in circulation, rigors, change in mentation, agitation, pain, unexpected trajectory, patient indicating they are feeling unwell, subjective nurse observation and nurse convinced that something is wrong without a rationale (Table 4).

**Table 3:** Signs and symptoms underlying worry or concern as indicator of deterioration, reported by nurses or analyzing RRS calls

10 indicators	Analysis qualitative studies (exploring cues nurses use)	Analysis qualitative studies (process of recognition)	Analysis RRS worry calls
Change in Breathing	Inability to talk in sentences, noisy breathing, gasping, wheezing, using accessory muscles, change in breathing [26], short of breath [26], breathless [27], increasing supplemental O2 to maintain SaO2, increase in respiratory rate (just more than the day before) [26] and low SpO2 [27]	Respiratory distress [36], breathing more labored, trouble breathing [38] and continued use of oxygen [11]	Dyspnea [20, 21], respiratory distress [23, 24], low SpO2 [24] and fall in SaO2 [23]
Change in Circulation	Impaired cutaneous perfusion [26], cold feet [26], coldness [27], tachycardia [27], (new) sweating [26, 27, 39], clammy [27, 39], (quite) pale [27], new observation, just a bit paler [26], color drainage changes, dusky [27], more pale than usual, porcelain pale, just a sort of gray, loss of pink color to their skin and color draining [27]	Cold feet [38], (new) sweating [11, 20], clammy [11], any change in color from patient’s usual one [11, 37], (quite) pale [11], pale gray, blue [11], ashen gray, sallow, change in skin color [38] and gray [31]	Arrhythmia [20], rhythm disturbance and hypertension [23]
Temperature			Fever [20], rigors, febrile [23] and hypo/hyperthermia [24]
Change in Mentation	Confused [26], impaired mentation [26], change in mentation [26], lethargic [26, 34], vaguer, slower [26], sleepy, not making sense, less verbal [34] and sensory change in the level of consciousness [34]	Withdrawn [11], confused [11, 38], drowsy [11, 37], lethargic [31] and sensory change in the level of consciousness [38]	Confused [23], drowsy [23], (mental) deterioration [24] and sensory change in the level of consciousness (without a decrease in Glasgow Coma Scale of ≥2 points) [20]
Agitation	Agitation [26], not getting out of bed [26], uneasy, want to sit in chair instead of bed, cannot get right position, restless [26], not comfortable [26], not comfortable in or out of bed, sitting on the edge of the seat, unsettled, distressed, anxious, climbing about, wanting tablets [27], pulling catheters and tubes out, calling out, pressing the buzzer more often [27], activity level [33] and increase activating the bed alarm [34]	Slumped in chair [11], not getting out of bed [11], not comfortable [38] and panicky [35]	Agitation [23, 24] restless [24] and aggression [21]
Pain	New or increasing pain, and jaw, neck, shoulder [26] chest [26] pain combined with bleeding [27]	(Unusual) pain [38]	Chest pain [20–23] and headache [20, 24]
Unexpected Trajectory	Not progressing [26], not expected trajectory, not following recovery pattern, not responding to treatment [26], abdominal distension, not eating [26] and bleeding [27]	Not progressing [11], abdominal distension, not eating [11] and vomiting [11]	Nausea [21, 24], vomiting [24], bleeding [22, 23], hypoglycaemia, dizzy [22–24], unstable blood sugars [21], syncope, collapse, fall [24] and seizures [22]
Patient indicates feeling unwell	Feeling of impending doom [27], feeling not right, feeling unwell [26, 27], new symptom, feeling different, feeling terrible, knowing something is happening, cannot explain what is wrong, generally unwell [26], scared and patient is not like this normally [27]		
Subjective nurse observation	Patient looks unwell [27], cannot settle the patient down, new symptom [26], does not look or seem right [27, 34], a look in the eyes, like a gaze [27], something is not right [39], patient looks terrible [33] and not patient’s normal face [34]	Reduced motivation, neglect, not getting out of bed, not acting in their normal way [38], patient looks unwell [11], changes in mood [38], does not look or seem right [35, 37, 38], something is not right [30 ,35], patient somehow looks so ill, difference in behavior, patient is quieter, patient did not open eyes [38] and patient looks really bad [31]	
Knowing without a rationale	Gut feeling [27], knowing something is happening, unconscious something [26], knowing something is wrong [26, 27, 39], intuition, sixth sense [37], cannot put a finger on it [27], just a feeling [27] and something does not look right [34]	Instinct [11], just knowing [11, 30], gut feeling [11, 38, 40], knowing something is wrong [37, 40], intuition, sixth sense [39], just a feeling [38], something does not look right [40], not as expected, cannot put a label on it [40], something is a tiny bit worse [40] and sensing [36]

**Table 4:** Thirty seven signs and symptoms underlying worry summarized in 10 indicators

Indicator	Underlying signs and symptoms
Change in breathing	Noisy breathing and/or short of breath and/or no full sentences and/or accessory muscles and/or increasing supplemental O2 to maintain SaO2 and/or increase in respiratory rate
Change in circulation	Colour and/or clammy and/or coldness and/or impaired perfusion and/or colour drainage changes and/or hypertension and/or arrhythmia
Temperature	Rigors and/or fever and/or hypothermia
Impaired mentation	Lethargic and/or confused and/or sensory change in level of consciousness
Agitation	Restless and/or anxious
Pain	New pain and/or increasing pain
No progress	No progress and/or abdominal distension and/or nausea and/or bleeding and/or dizzy and/or fall and/or hypoglycaemia
Patient	Not feeling well and/or feeling of impending doom
Subjective nurse observation	Change in behaviour and/or does not look good and/or a look in the eyes, like a gaze
Knowing without a rationale	Gut feeling and/or knowing something is wrong

Qualitative studies described up to nine different indicators, that is, all except rigors [25–27, 30, 33–40]. The analysis of the worry calls yielded up to seven different indicators, that is, all except for the three indicators: patient, nurse observation and knowing without a rationale [20–24]. Table 5 presents an overview of the different indicators in the studies. Both qualitative and quantitative studies mention deteriorating vital signs, like a fall in SaO2, hypertension, arrhythmia and fever [11, 20, 23, 24, 26, 27, 34, 38], as triggers for worry or concern. The majority of these studies report worry or concern based on minor changes in vital signs [20, 26, 27, 34, 38]; this was also reported in two other studies [31, 35].

**Table 5:** Frequency of indicators per study

	First author, year, reference	Change in breathing	Change in circulation	Temperature	Change in mentation	Agitation	Pain	Unexpected trajectory	Patient indicates feeling unwell	Subjective nurses observation	Knowing without a rationale	Number of indicators described per study
Analysis of qualitative studies	Andrews, 2005 [11]	x	x		x	x		x		x	x	7
	Cioffi, 2009 [26]	x	x		x	x	x	x	x	x	x	9
	Cioffi, 2000 [27]	x	x			x	x	x	x	x	x	8
	Cox, 2006 [30]									x	x	2
	Donaldson, 2009 [31]		x		x					x	x	4
	Endacott, 2007 [33]					x				x		2
	Gazarian, 2010 [34]				x	x				x	x	4
	Leach, 2010 [35]					x				x		2
	Massey, 2014 [36]	x									x	2
	McDonnell, 2013 [37]		x		x					x	x	4
	Minick, 2003 [38]	x	x		x	x	x			x	x	7
	Pattison, 2011 [39]		x							x	x	3
	Williams, 2011 [40]										x	1
Analysis of worry RRS calls	Boniatti, 2010 [20]	x	x	x	x		x					5
	Hourihan, 1995 [21]	x				x	x	x				4
	Laurens, 2011 [22]						x	x				2
	Parr, 2001 [23]	x	x	x	x	x	x	x				7
	Santiano, 2009 [24]	x		x	x	x	x	x				6
Total number of studies describing an indicator		9	9	3	9	10	8	7	2	11	11	

## Discussion

We examined signs and symptoms underlying worry or concern of nurses in relation to early recognition of deteriorating patients on general wards in acute care hospitals. Our most important finding is that 37 different signs and symptoms, summarized in 10 indicators, can alert nurses that a patient may rapidly deteriorate. Seven of the included studies reported the presence of worry or concern before vital signs worsened.

### Signs and symptoms underlying worry or concern

Although nurses find it hard to put intuition into words, we extracted objective signs and symptoms underlying worry or concern, or intuitive knowing. The indicators change in breathing, change in circulation, rigors and change in mentation can be related or precede deviating vital signs. Others are not related to vital signs: agitation, pain, unexpected trajectory and patient indicates feeling unwell. The indicator subjective nurse observations might partly cover the inability to explain what is wrong (patient does not look good), on the other hand it covers subtle signs such as change in behavior or the look in the patient’s eyes, both appealing to the observation skills of nurses. The indicator knowing without a rationale comprises the intuitive knowing that something is wrong based on possible unconscious observations. Skilled judges are often unaware of the cues that guide them [41]. Still, intuition plays an important and excepted role in nurses’ decision-making [42, 43]. Intuition is believed to develop over time [3], so less experienced nurses might have more problems or even not see or acknowledge the importance of signs. The overview of signs and symptoms can contribute to the awareness of the importance of the mentioned indicators, and either help make the unconscious awareness for expert nurses more objective, or help less experienced nurses to articulate their feelings. This will improve the communication regarding deteriorating patients who do not yet meet the RRS calling criteria.

The significance of some of the signs and symptoms we found as early signs of deterioration has already been demonstrated in other studies. Shortness of breath and chest pain were present before cardiac arrest (CA) [10]. Buist *et al.* [44] found significantly lower rates of CA and mortality after implementation of an RRS with respiratory distress, difficulty speaking, agitation or delirium, uncontrolled pain and failure to respond to treatment included as RRS calling criteria. Another study found a significant association between the following: poor peripheral circulation and mortality and CA; new pain with mortality and ICU admission; alteration in mentation with mortality, CA and ICU admission; uncontrolled pain with CA; and chest pain with CA and ICU admission [45]. The signs and symptoms underlying worry or concern that we found in the literature alert nurses, and as such motivate nurses to take action to verify their intuitive feelings, which makes them valuable as potential early indicators of deterioration. While the importance of these signs and symptoms has been highlighted in several studies, they are not included as such in most RRSs. The National Early Warning Score (NEWS), based on vital signs, discriminated more patients at risk of unplanned ICU admission or mortality than 33 other Track and Trigger Systems [46]. As the authors discuss, the NEWS must be seen as the minimum in monitoring patients, and should be used alongside other triggers such as worry or concern of nurses and other criteria.

### Implications for practice

The 10 indicators identified in our study might help nurses to articulate their worries or their intuition, and contribute to better communication on deterioration. Yet without a medical response, an opportunity would be missed to intervene in an early stage. The medical response indeed prevents patients from further deterioration. This implies that not only nurses should be aware of the importance of the indicators, but also that doctors should acknowledge their importance. RRSs that include worry as calling criterion do give nurses the opportunity to call, but still would benefit if nurses articulate their worries in objective words. The presence of worry or concern of nurses before vital signs deteriorate suggests that the signs underlying the worry or concern of nurses have potential as early indicators of deterioration, and could imply that in RRSs without the worry or concern criterion, the chances for early activation of the RRT are reduced.

### Limitations

This systematic literature review has several limitations. First, results from observational and qualitative designs are not considered strong in the hierarchy of evidence. However, due to the nature of research involved (exploratory or evaluating) more rigorous study designs would not have been appropriate. Second, the heterogeneity of studies prevented the conduct of another type of analysis other than a content analysis and thematic synthesis, reducing evidence strength; however we consider these studies valuable to initiate more rigorous research. Third, the majority of included studies did not focus primarily on worry or concern, therefore worry or concern could have been present more often than documented in these studies. Fourth, most studies included had quality weaknesses, but we feel that the recurrence of similar findings in both quantitative and qualitative studies support the observations, especially with regard to our proposed indicators. Last, the instrument for quality assessment of quantitative studies has not been validated, yet the items used for assessment were all relevant for internal validity.

## Conclusions

We found 37 signs and symptoms summarized in 10 general indicators reflecting the nature of nurses’ worry or concern. Nurses may incorporate these signals in their assessment of patients and the decision to call for assistance. Nurses’ subjective feeling of worry or concern is valuable in the process of recognizing deteriorating patients in general wards. Its presence even before vital signs have changed suggests potential for improving care in an early stage of deterioration. However, the number of studies is limited. The evidence found in this review was merely from retrospective research, which might have biased the results. A prospective cohort study is warranted, with nurses recording the indicators and worry or concern systematically, to establish if and how worry or concern can improve the existing calling criteria in RRSs. Potentially, this may lead to earlier recognition and treatment of deteriorating patients and improve patient outcomes.

## Key messages

- A total of 10 indicator domains describe the nature of worry or concern of nurses.
- Variable incidences and combinations of indicator domains scored are encountered among deteriorating patients in general wards.
- Nurses frequently describe worry or concern before changes in vital signs occur, suggesting potential relevance as early indicator of deterioration.
- As study designs were merely retrospective, prospective evaluations are warranted to assess the value to clinical relevance of worry or concern of nurses

## Additional files

Additional file 1:
**Inclusion and exclusion criteria.**
Additional file 2:
**Full PubMed search.**
Additional file 3:
**Study characteristics.**

## Abbreviations

CA: Cardiac Arrest
ICU: Intensive Care Unit
RRSs: Rapid Response Systems
RRT: Rapid Response Team
STROBE: Strengthening the Reporting of Observational Studies in Epidemiology
UK: United Kingdom
USA: United States of America
