# Supplementary material for: Nurses’ worry or concern and early recognition of deteriorating patients on general wards in acute care hospitals: a systematic review
Source: Crit Care. 2015 May 20;19(1):230. doi: 10.1186/s13054-015-0950-5 (PMC4461986; doi:10.1186/s13054-015-0950-5)
Supplement: Additional file 1: — Inclusion and exclusion criteria. [file 13054_2015_950_MOESM1_ESM.pdf]

Additional file 1 In- and exclusion crirteria

Inclusion criteria	Original studies, all designs and languages
	Studies performed in general wards in acute care hospitals
	Studies concerning adult patients (age 18 years and older)
	Studies that address ‘worry/concern’ of nurses in the process of recognition of deterioration in patients
	Studies that address ‘worry/concern’ of nurses, preceding the calling for assistance/activation of the RRT
	Full-text was available
Exclusion criteria	Studies that focused solely on specialized wards such as Emergency Department, Intensive Care Unit, Medium Care Unit, Obstetrics ward, Operating Room, Paediatric wards, Psychiatry wards
	Studies concerning homecare
	Studies of low methodological quality
