# Supplementary material for: Nurses’ worry or concern and early recognition of deteriorating patients on general wards in acute care hospitals: a systematic review
Source: Crit Care. 2015 May 20;19(1):230. doi: 10.1186/s13054-015-0950-5 (PMC4461986; doi:10.1186/s13054-015-0950-5)
Supplement: Additional file 2: — Full PubMed search. [file 13054_2015_950_MOESM2_ESM.pdf]

Additional file 2 PubMed Search n=2555

Search ((((((((((((("nurses"[MeSH Terms] OR "nurses"[tiab])) OR (nurse[tiab])) OR ("nursing"[MeSH Terms] OR "nursing"[tiab])) OR ("nursing staff"[MeSH Terms]))))))) AND ((((((((("reflective thinking"[tiab])) OR ("reflective reasoning"[tiab])) OR ("non analytical reasoning"[tiab])) OR ((((((((((((((("intuition"[MeSH Terms] OR "intuition"[tiab])) OR (intuitive[tiab] OR intuitiveness[tiab])) OR ("nursing diagnosis"[MeSH Terms] OR "nursing diagnosis"[tiab])) OR ("nursing assessment"[MeSH Terms] OR "nursing assessment"[tiab])) OR ("observation"[MeSH Terms] OR "observation"[tiab] OR observations[tiab])) OR ("judgment"[MeSH Terms] OR "judgment"[tiab] OR "judgement"[tiab])) OR ("recognition (psychology)"[MeSH Terms] OR "recognition"[tiab] OR "early recognition"[tiab] OR recognize[tiab] OR recognise[tiab])) OR ("decision making"[MeSH Terms] OR "decision making"[tiab])) OR ("cues"[MeSH Terms] OR "cues"[tiab])) OR ("gut feeling"[tiab] OR "gut feelings"[tiab] OR "clinical gaze"[tiab] OR "nursing gaze"[tiab])) OR (knowing[tiab] OR concern[tiab] OR concerned[tiab] OR "changes of concern"[tiab] OR "concerned about a patient"[tiab])) OR (worry[tiab] OR worried[tiab] OR worrisome[tiab])) OR ("doesn't look right"[tiab])) OR ("Unexplained onset of agitation"[tiab]))))))) OR "triggers"[tiab])) AND ((((((((((((("emergencies"[MeSH Terms] OR "emergencies"[tiab])) OR ("critical illness"[MeSH Terms] OR "critical illness"[tiab] OR "hospital rapid response team"[MeSH Terms] OR "hospital rapid response team"[tiab])) OR ("rapid response team"[tiab] OR "rapid response teams"[tiab] OR "medical emergency team"[tiab] OR "medical emergency teams"[tiab])) OR ("outreach team"[tiab] OR "outreach teams"[tiab] OR "emergency team"[tiab] OR "emergency teams"[tiab])) OR ("emergency assistance"[tiab] OR "rapid response system"[tiab] OR "rapid response systems"[tiab])) OR (deteriorate[tiab] OR deteriorated[tiab] OR deterioration[tiab] OR deteriorations[tiab])) OR ("deteriorating patient"[tiab] OR "deteriorating patients"[tiab] OR worsening[tiab] OR "critically ill"[tiab])) OR ("patient problem"[tiab] OR "patient problems"[tiab] OR "critical conditions"[tiab] OR "patient at risk"[tiab] OR "patients at risk"[tiab])) OR ("at risk patient"[tiab] OR "at risk patients"[tiab] OR "early warning score"[tiab] OR "alarm score"[tiab] OR "track and trigger "[tiab])))) Filters: Publication date from 1900/01/01 to 2014/01/31
