# Supplementary material for: Nurses’ worry or concern and early recognition of deteriorating patients on general wards in acute care hospitals: a systematic review
Source: Crit Care. 2015 May 20;19(1):230. doi: 10.1186/s13054-015-0950-5 (PMC4461986; doi:10.1186/s13054-015-0950-5)
Supplement: Additional file 3: — Study characteristics. [file 13054_2015_950_MOESM3_ESM.pdf]

Additional file 3 Study characteristics

Author	Design	Aim	Data collection	Sample	Setting	RRS	Outcome measures used
Andrews and Waterman, 2005	Grounded theory Theoretical sampling until saturation	Investigate difficulties ward staff experienced in detecting deterioration and how it is resolved	Interviews (open ended quetions) Participant and nonparticipant observation	30 Nurses, 7 doctors, 7 healthcare support workers	1 surgical and 1 general medical ward University teaching hospital (UK)	Type RRT: outreach Aggregated scoring system No "worry/concern" criterion	Signs and symptoms of recognition of deterioration
Boniatti et al. 2010	Observational Cross-sectional	Describe reasons for RRS activation and verify association of calling criteria with 30 day mortality	Review registration of RRS-calls	1051 RRS-calls for 901 patients during January 2007 to June 2008	Type of ward: not specified 794 bed university-affiliated hospital (Brasil)	Type RRT: MET Single-parameter system Criterion "worry/concern" included	Analysis "worry/concern" criterion
Cioffi et al. 2009	Exploratory descriptive Purposive and snowball sampling Analysis procedures according to Klein et al.1989	Identify cues of potential early clinical deterioration used to recognise a patient of concern who is not meeting the current objective calling criteria	Interviews; recall of incident, use of probes	17 Nurses	12 medical , 2 surgical, 3 HDP units 2 tertiary referral hospitals and 2 metropolitan hospitals (Australia)	Not reported	Signs and symptoms of recognition of deterioration
Cioffi 2000	Exploratory Descriptive Purposive sample	Explore patient characteristics and process of recognition nurses when worried.	Unstructured interviews, describing an experience	32 Nurses > 5 years experience, high RRS users	Surgical, renal, gynaecological, CCU, orthopedics, with high numbers of RRS calls. A teaching hospital and a peripheral hospital (Australia)	Type RRT: MET Single-parameter system Criterion "worry/concern" included	Signs and symptoms of recognition of deterioration
Cox et al. 2006	Exploratory Descriptive Purposive sample Content analysis	Explore factors that influence experiences of trained nurses caring for critically ill patients	Semi-structured interviews; recount an incident	7 Nurses Experience: newly qualified to 20 years.	1 medical ward District general hospital (UK)	Not reported	Signs and symptoms of recognition of deterioration
Donaldson et al.2009	Mixed-method qualitative part for review: Descriptive Convenience sample Thematic analysis until saturation	Evaluate impact of RRT(from nurse perspective)	Interviews; open-ended questions	56 Nurses	Acute care units 136 – 412 beds; 9 robust RRT adopter hospitals and 9 delayed RRT adopter hospitals (USA)	Type RRT: RRTnurse led Calling system not reported	Signs and symptoms of recognition of deterioration
Endacott et al. 2007	Mixed-method qualitative part	Identify cues ward nurses/doctors use to	Semi-structured interviews	14 Doctors and 11 nurses caring for 17 patients 24 hours before	General wards 220 bed regional hospital	Not reported	Signs and symptoms of recognition of deterioration

	for review: Descriptive Purposive sample Content analysis	identify patient deterioration Examine assessment and communication of deterioration in patients on acute wards		unexpected ICUadmission	(Australia)		
Gazarion et al. 2010	Descriptive Purposive sampling until saturation Cognitive task	Describe cues and factors employed by nurses to identify and interrupt a potential preventable CPA	Interviews	13 Nurses (10 RN's , 1 Nurse in Charge, 2 staff nurses unit perceptors) caring for patients who had experienced a prearrest period)	4 medical wards 747 bed academic medical center (USA)	Not reported	Signs and symptoms of recognition of deterioration
Hourihan et al. 1995	Observational Descriptive prospective cohort	Describe utilisation of RRS after implementation	Registration of RRS-calls	294 RRS-calls from April - October 1994	All wards, 460 bed university teaching hospital (Australia)	Type RRT: MET Single-parameter system Criterion "worry/concern" included	Analysis of RRS calls
Laurens & Dwyer, 2010	Quasi-experimental, Before and after study	Determine effect RRS implementation on mortality rates, cardiopulmonary arrests, and ICU-admissions	Retrospective patient files Prospective RRS registration	105 activations of cardiac arrest team pre-intervention period 296 RRS activations	All wards, 150 bed regional teaching hospital (Australia)	Type RRT: MET Single-parameter system Criterion "worry/concern" included	Analysis "worry/concern" criterion
Leach et al. 2010	Grounded theory Purposive sampling Open coding, constant comparison and contrasting	Investigate how RNs rescue patients in hospitals with RRT	Semi-structured interviews	50 Nurses involved with RRT's (14 bedside staff RNs who had called RRTs,16 RRTstaff RNs, 2 respiratory therapists who had responded to RRTs, 8 nurse supervisors who had observed RRTs)	Type of ward: not mentioned 6 acute care hospitals (non-profit community, magnet-designated, public, academic, for-profit community and integrated delivery system) (USA)	Type RRT: RRT Calling system not reported;	Signs and symptoms of recognition of deterioration
Massey et al. 2013	Interpretive qualitative approach Transcribed verbatim and thematical analysis	Explore nurses' experiences and perceptions of using and activating a RRS	In-depths semi-structured interviews.	15 Registered ward nurses	General wards. Large public teaching hospital (Australia)	Type RRT: MET Single-parameter system Not specifically mentioned whether 'worry/concern' was calling criterion	Signs and symptoms of recognition of deterioration
McDonnell et al. 2012	Mixed-method qualitative part for review: Descriptive Purposive sampling Thematic framework analysis (Ritchie/Spencer 1994)	Evaluate impact of new T&T and observation charts on the knowledge and confidence of nurses to recognize and manage deteriorating patients	Semi structured interviews	15 Registered nurses	Type of ward: surgical, orthopaedic, acute medicine, Medicine District general hospital > 500 beds (UK)	Type RRT: outreach Aggregated scoring system No "worry/concern" criterion.	Signs and symptoms of recognition of deterioration
Minick	Interpretative	Describe phenomenon	In-depths group	14 Nurses	Orthopaedics, neurology, renal, oncology,	Not reported	Signs and symptoms of

andHarvey 2003	Phenomenology Purposive sampling	of early problem recognition among medical-surgical nurses	interviews, describing an experience		cardiac, or pulmonary ward Urban hospital (USA)		recognition of deterioration
Parr et al. 2001	Observational Cross-sectional	Describe reasons for, and immediate outcome following RRS activation	Review registration of RRS-calls	713 RRS- calls concerning 559 patients during the year 1998	Wards not specified, ED excluded 580 bed tertiary teaching university-affiliated hospital (Australia)	Type RRT: MET Single-parameter system Criterion "worry/concern" included	Analysis of "worry/concern" RRS-calls
Pattison & Eastham, 2011	Mixed-method qualitative part for review: Explanatory Theoretical sampling Using grounded theory principles	Explore referrals (characteristics) to CriticalCareOutreachTeam(CCOT)	In-dephts interviews, loosely structured	7 nurses and 2 doctors	Patients with cancer referred to CCOT. Specialist hospital over an 8 months period (UK)	Type RRT: outreach Aggregated scoring system Not specifically mentioned whether 'worry/concern' was calling criterion.	Signs and symptoms of recognition of deterioration
Santiano et al. 2009	Observational Cross-sectional	Explore reasons nurses use subjective 'worried' calling criterion. Compare outcomes of worried with calls on objective criteria	Review registration of RRS-calls	3189 RRS calls during 2006	Type of ward: not specified 6 acute care hospitals (tertiary referral centre, major metropolitan, metropolitan and rural) (Australia)	Type RRT: MET Single-parameter system Criterion "worry/concern" included	Analysis of "worry/concern" RRS-calls
Williams et al. 2011	Descriptive Convenience sample Content analysis	Clarify nurse perceptions of RRT's	Focus groups, 15 item topic guide	13 nurses	156 bed community hospital General wards (USA)	Not reported	Signs and symptoms of recognition of deterioration
